# Prevalence and correlates of depressive symptoms among high school adolescent girls in southern Uganda

**DOI:** 10.1186/s12889-020-09937-2

**Published:** 2020-11-25

**Authors:** Proscovia Nabunya, Christopher Damulira, William Byansi, Joelynn Muwanga, Ozge Sensoy Bahar, Flavia Namuwonge, Eloho Ighofose, Rachel Brathwaite, Wilberforce Tumwesige, Fred M. Ssewamala

**Affiliations:** Washington University in St. Louis, Brown School of Social Work, International Center for Child Health and Development (ICHAD), One Brookings Drive, St. Louis, MO 63130 USA

**Keywords:** Depressive symptoms, Prevalence, Adolescent girls, Uganda, Mental health

## Abstract

**Background:**

In sub-Saharan Africa (SSA), adolescent girls and young women are three times more likely than boys to have depressive disorders. Understanding adolescents’ unique and common vulnerabilities and protective factors is essential for the development of appropriate interventions and programming focused on child and adolescent mental health. This paper examines the prevalence and predictors of depressive symptoms among high school adolescent girls in southern Uganda.

**Methods:**

Baseline data from a longitudinal cluster randomized study involving 1260 adolescent girls (14–17 years), recruited from 47 secondary schools were utilized. Depressive symptoms were estimated using the 21-item Beck’s Depression Inventory. Hierarchical linear regression modelling was utilized to estimate key predictors of depressive symptoms among adolescent girls.

**Results:**

Of the total sample, 16.35% (*n* = 206) reported severe depressive symptoms and almost one in every three adolescent girls interviewed (29.68%, *n* = 374) reported moderate symptoms. These symptoms were more prevalent among older adolescents (16 years and above). In addition, family relationships, social support, as well as measures of psychological wellbeing (self-concept and self-esteem) were all associated with lower levels of depressive symptoms. Hopelessness was associated with higher levels of depressive symptoms among adolescent girls.

**Conclusion:**

Findings from this study indicate a high prevalence of depressive symptoms, especially among older adolescent girls. In addition, family support factors and adolescents’ psychological wellbeing were associated with low levels of depressive symptoms –pointing to the need to strengthen family functioning and adolescent’s psychological wellbeing to mitigate risks. Taken together, findings support increasing calls for early screening and detection of depressive symptoms to facilitate timely referral to care and treatment. Findings may also inform the development and incorporation of gender-specific mental health components in programming targeting adolescent girls, in low-resource communities in SSA.

**Trial registration:**

This trial was prospectively registered with ClinicalTrials.gov (registration number: NCT03307226) on 11 October 2017.

## Background

Mental health disorders account for 16% of the global burden of disease and injury among children and adolescents between 10 and 19 years of age [1]. Depression is one of the leading causes of illness and disability among adolescents between 10 and 14 years [1, 2]. In sub-Saharan Africa (SSA), there is limited epidemiological data on the prevalence of depression in young people [3, 4]. Additionally, there is poor understanding of mental health illness, that disorders such as depression are unrecognized and as such, remain untreated [5, 6]. On the other hand, studies document considerable levels of other mental health problems among children and adolescents [7, 8]. It is estimated that 1 in 7 adolescents have significant mental difficulties, with 1 in 10 having a specific psychiatric disorder in SSA [3]. Yet, there are fewer economic and human resources dedicated to the mental health of children and adolescents in the region [9]. Given that most mental health disorders start early in childhood and continue to rise through adolescence [10–13], if unaddressed, these conditions extend to adulthood resulting in poor health outcomes, limiting opportunities for a productive adult life [13, 14].

### Risk and protective factors

Understanding adolescents’ unique and common vulnerabilities to depression, as well as protective factors is essential for the development of appropriate interventions and programming focused on child and adolescent mental health. Previous studies have documented the disproportionate prevalence of depression and other mental health disorders among females compared to males [15–19]. Socio-economic factors including poverty, gender-based violence, sexual exploitation, and exclusion from decision-making processes increase susceptibility and exposure to mental health risks among adolescent girls and limit their ability to seek, access and use mental health services [20–22]. These factors combine with biological, emotional and cognitive processes associated with puberty [23, 24], to further increase the risk of depression among adolescent girls.

In addition, negative thoughts associated with depression influence both individuals’ feelings about themselves and their behaviors [25, 26], and can lead to unhealthy decision making [27]. Depressed adolescents are more likely to have low educational attainment, be unemployed and at high risk for death due to suicide [28, 29]. In addition, adolescents experiencing depression and other mental health issues are more likely to engage in risky behaviors, including unprotected sexual activity, having multiple sexual partners [30–32] and substance abuse [28]. Indeed, depressive symptoms are associated with cumulative HIV incidence among adolescent girls and young women in SSA [33].

A number of individual level and family-level factors have been documented as protective against depressive symptoms. At the individual level, self-esteem has an inverse relationship with depression [34–38], and adolescents with low self- esteem are more likely to be depressed later in life [38–40]. Similar findings have been documented for hopelessness [41, 42]. In addition, the relationship between family and community social capital and mental health outcomes among children and adolescents has been widely documented [43–46]. Social capital in the form of social support, especially from parents/caregivers and family members is consistently associated with adolescent’s protection from depression [47–50]. Adolescents reporting high levels of family cohesion, trust and closeness also report better mental health and behavioral outcomes [51–53]. Moreover, parent–child relationships characterized by social support, positive communication, nurturance, parental involvement and low levels of conflict are associated with fewer reported mental health issues among children and adolescents [53–57]. Thus, interventions that target both individual level assets, such as self-esteem and future orientation, as well as family-level resources, including social support to improve the overall mental health functioning of adolescents as they transition into adulthood, are highly critical.

The above studies have mainly been conducted in high income countries. Consistent with other countries in SSA, few studies in Uganda have documented the prevalence of depressive symptoms among young people [58, 59], and among adolescent girls in particular. Available studies have mainly focused on highly vulnerable children and adolescents, such as those orphaned by HIV/ AIDS [60–65], those with chronic conditions [66–70], and traumatized adolescents in war torn and post-conflict settings [71, 72]. To our knowledge, only one study has focused on school-going adolescents (14–17 years) in a non-conflict region [73]. Findings from this study indicate prevalence rates of 21% on the child depression inventory measure, with higher prevalence among adolescent girls compared to boys. This study was however conducted in the central region of Uganda, with a sample of 591 adolescents, within a mix of boarding schools and single sex schools, with slightly different socio-economic status of the students’ body, compared to the general student population in Uganda. In addition, this study did not assess social support –which has been documented as an important predictor of mental health wellbeing among individuals, including children and adolescents [43–45, 74].

Thus, this study aims to add to existing limited literature and to address gaps in the prevalence rates and predictors of depressive symptoms among high school adolescent girls in Uganda. More specifically, this study aims to address the following research questions: 1) What are the prevalence rates of depressive symptoms among adolescent girls in southern Uganda, a geographical region heavily impacted by HIV/AIDS? 2) Do levels of depressive symptoms vary based on age groups i.e., younger versus older adolescent girls? 3) What are the key individual and family -level predictors of depressive symptoms among adolescent girls? This is an important area of inquiry, as findings may inform the development of and incorporation of gender-specific mental health components in programs and policies targeting adolescent girls –given the high levels of vulnerability, especially in low-resource communities in SSA. Moreover, findings may point to the timing of screening and intervening for depressive symptoms among adolescents.

## Methods

### Study sample

This study utilized baseline data from the *Suubi4Her* study, a longitudinal randomized clinical trial (2017–2022), funded by the National Institute of Mental Health (Grant # R01MH113486). A total of 1260 adolescent girls between 14 and 17 years of age at study initiation, were enrolled in the study. Adolescents were eligible to participate if they met the following inclusion criteria: 1) female, 2) age 14–17 years, 3) enrolled in the first year of secondary school, and 4) living within a family (broadly defined and not an institution or orphanage).

### Participant recruitment

Adolescents were identified and recruited from 47 secondary schools in five geopolitical districts in southern Uganda. All schools in the study were matched based on socioeconomic status of the students attending these schools, school size (total number of students enrolled), location (urban vs. rural), and overall performance based on the Uganda Certificate of Education (UCE) examinations, administered by the Uganda Government’s Ministry of Education and Sports. School administrators helped to identify potential participants and their parents/caregivers. Parents/caregivers were given flyers notifying them of the study and inviting them to contact the school headteacher for further details. In addition, community development officers and implementing partners distributed flyers during their frequent community visits to inform parents/caregivers whose children met the inclusion criteria but may not yet have reported to school. Caregivers and adolescents who expressed interest were later invited to meet with the in-country project coordinator for a one-on-one informational meeting. During the meeting, parents/caregivers and adolescents were informed verbally and in writing, the purpose of the study, voluntary participation, extent of their participation, risk and benefits, as well as protection and confidentiality issues. Interested caregivers/parents signed the informed consent and adolescent girls signed the assent forms. Detailed information on participants recruitment and selection process, power analysis, as well as the intervention is described in the study protocol and in our other publications [75, 76].

### Data collection and measures

Detailed data collection procedures are provided elsewhere [75, 76]. In brief, a 90-min interviewer -administered survey was utilized to collect data. Interviews were conducted in the Luganda local language by trained Uganda interviewers. All measures utilized in this study were tested in our previous studies in Uganda among children and adolescents affected by HIV/AIDS in the study area [62–65, 77, 78].

#### Outcome variable

Depressive symptoms were measured using the Beck’s Depression Inventory (BDI) [79]. The 21-item scale measures characteristic attitudes and symptoms of depression ranked based on severity (0 = least, 3 = most). Scores range from 0 to 63 (alpha =0.83), with higher scores indicating higher levels of depressive symptoms.

#### Measures of psychological wellbeing

*Self-concept* was measured using the Tennessee Self-Concept Scale [80]. The 20-item scale measures children’s perception of identity and self-satisfaction (1 = always false and 5 = always true). The scale ranges from 20 to 100 (alpha =0.83), with higher scores indicating higher levels of child self-concept. Participants’ *self-esteem* was measured using the Rosenberg Self-Esteem Scale [81]. The 10-item scale measures individual self-esteem (1 = strongly disagree to 4 = strongly agree). Scores range from 10 to 40 (alpha = 0.66), with higher scores indicating high levels of self-esteem. *Hopelessness* was measured using the Beck Hopelessness Scale [82]. The 20-item scale measures children’s hopelessness and pessimistic attitudes toward the future (1 = true and 0 = false). Scores range from 0 to 20 (alpha =0.71), with higher scores indicating higher level of hopelessness and pessimistic attitudes.

#### Family and social support factors

*Family cohesion* was measured using 7 items that assess family members’ commitment and support for each other (1 = never and 5 = always) [83, 84]. Scores range from 7 to 35 (Cronbach alpha = 0.72), with high scores indicating higher levels of family cohesion. Participants’ *perceived caregiver support* was assessed using 17 items adapted from the Social Support Behaviors Scale (SS-B) scale (1 = never and 5 = always) [85]. Scores range from 17 to 85 (alpha =0.78), with high scores indicating high levels of perceived support from caregivers. *Family care and relationship* was measured using 6 items related to things that parents sometimes do with their children (1 = never and 5 = always). Scores ranges from 6 to 30 (alpha = 0.60), with higher scores indicating higher levels of family care and relationships. *Social support from multiple sources* was measured using 30 items adapted from the Friendship Qualities Scale [86]. Items assess the impressions of the quality of children’s friendships and relationships (1 = never and 5 = always). Scores range from 30 to 150 (alpha =0.81), with high scores indicating higher levels of social support and relationships.

Finally, participant’s sociodemographic and household characteristics included in the analysis as covariates were: 1) participants’ age categorized into 14–15 years versus 16–17 years, 2) orphanhood status, 3) primary caregiver, 4) household size, and 5) household assets.

### Data analysis procedures

We analyzed sociodemographic and household characteristics of the sample, followed by bivariate analyses of predictors of depressive symptoms (sociodemographic and household characteristics, family and social support factors and psychological wellbeing) across participants’ age groups. We estimated the chi- square or t-test values for each of the variables. To address research questions 1 and 2 (i.e. determine the prevalence of depressive symptoms and variation by age groups), BDI scores were divided into 4 categories based on the scoring guidelines in non-clinical populations [87], minimal (0–9), mild (10–18) moderate (19–29) and severe (30+) symptoms. We then conducted chi square tests by age groups i.e., younger adolescents (14-15 years) versus older adolescents (16–17 years). Older adolescents tend to have high levels of depressive symptoms compared to young ones [10, 11]. To address research question 3, a summated score of the BDI scale was created. Hierarchical regression models were conducted to determine the predictors of depressive symptoms. We used the Variation Inflation Factor (VIF) to check for multicollinearity of the predictors and all the mean values were less than 5. We conducted three models, with each model controlling for a block of predictors. Model 1 controlled for socio-demographics and household characteristics; model 2 controlled for family and social support factors, and model 3 controlled for psychological wellbeing. We compared the adjusted R squares to determine the strength of each model. Data was analyzed in STATA version 15.

## Results

### Sample characteristics

Baseline sample characteristics are presented in Table 1 below. The average age of participants is 15.4 years, with the majority of participants (57%) between 14 and 15 years. About 83% of participants are non-orphan, and 76.6% identified a biological parent as their primary caregiver. The average household is 7 people, with three children under the age of 18 years living in the household. The average score on family asset index (including land, modes of transportation and communication, gardens, farm animals and small business) is 11.46 out of the expected 21, indicating moderate levels of asset ownership.

**Table 1:** Sample Characteristics

Variable	Total Sample (*N* = 1260) *% (n)*
*Age*	
14 to 15 years	56.98 (718)
16 to 17 years	43.02 (542)
*Orphan hood Status*	
Orphan	17.06 (215)
Non - orphan	82.94 (1045)
*Primary Caregiver*	
Biological parent	76.59 (965)
Grandparent	11.11 (140)
Other relative	12.30 (155)
*Household size (Mean, SD)*	
Number of people in HH	7.00 (2.71)
Number of children in HH	3.49 (2.10)
Family Assets (*Mean, SD)*	11.46 (3.26)

#### Participants’ sociodemographic and household characteristics, family and social support factors and psychological wellbeing, by age group

Results from bivariate analyses for participants’ sociodemographic and household characteristics, family support, social support factors and psychological wellbeing by age groups are presented in Table 2. Participants in both age groups were similar in terms of orphanhood status and primary caregiver reports. Older adolescents were more likely to report on average, slightly more children living in the household compared to younger adolescents (3.64 versus 3.39 children). The mean difference between the two age groups was statistically significant (*t* = − 2.02, *p* ≤ .05). In terms of social support factors, younger adolescents reported slightly higher scores on all measures of family and social support factors compared to older adolescents, including on family cohesion (mean = 26.61 versus 26.53), family care and relationships (mean = 25.28 versus 24.01) and perceived child-caregiver support (mean = 57.16 versus 56.44). The mean score difference on the measure of family care and relationship was statistically significant (*t* = 5.27, *p* = .001). In addition, younger adolescents were more likely to report slightly higher scores on social support from parents/guardians (*t* = 2.67, *p* ≤ .01) and from teachers (*t* = 2.58, *p* ≤ .01) compared to older adolescent. Similar results were observed on measures of psychological wellbeing. Specifically, younger adolescents reported slightly higher scores on self-concept (mean = 81.79 versus 79.58) and self-esteem (mean = 34.20 versus 33.75), and lower scores on measures of hopelessness (mean = 3.96 versus 4.53). The mean score difference between the two age groups for both self-concept (*t* = 3.26, *p* = .001) and hopelessness (*t* = − 3.41, *p* = .001) were statistically significant.

**Table 2:** Bivariate Analysis Results: Sociodemographic and household characteristics, family and social support factors, and participants’ psychological wellbeing by age groups

Variable	Total Sample (*N* = 1260) Mean (SD)	14–15 years (*n* = 718) Mean (SD)	16–17 years (*n* = 542) Mean (SD)	χ^2^/t-test
*Orphan hood Status (%, n)*				
Orphan	17.06 (215)	17.27 (124)	16.79 (91)	0.05
Non - orphan	82.94 (1045)	82.72 (594)	83.21 (451)	
*Primary Caregiver (%, n)*				
Biological parent	76.59 (965)	75.91 (545)	77.49 (420)	1.88
Grandparent	11.11 (140)	10.72 (77)	11.62 (63)	
Other relative	12.30 (155)	13.37 (96)	10.89 (59)	
*Household size*				
Number of people in HH (min/max: 2–31)	7.00 (2.71)	6.88 (2.78)	7.16 (6.94)	−1.81
Number of children in HH (min/max: 0–13)	3.49 (2.10)	3.39 (2.01)	3.64 (3.45)	−2.02*
Family assets (min/max: 0–20)	11.46 (3.26)	11.32 (3.29)	11.64 (3.21)	−1.71
*Family support factors*				
Family cohesion (min/max:7–35)	26.58 (5.69)	26.61 (5.73)	26.53 (5.66)	0.25
Family care and relationship (min/max: 9–30)	24.74 (4.27)	25.28 (4.09)	24.01 (4.41)	5.27***
Perceived child-caregiver support (min/max: 29–81)	56.85 (6.73)	57.16 (6.72)	56.44 (6.72)	1.85
*Social support from multiple sources*				
Guardian (min/max: 9–30)	23.66 (4.19)	23.93 (4.12)	23.29 (4.25)	2.67**
Classmate (min/max: 7–25)	17.78 (3.52)	17.87 (3.55)	17.67 (3.47)	1.01
Teacher (min/max:10–30)	22.85 (4.23)	23.11 (4.22)	22.49 (4.21)	2.58**
Friend/peer (min/max: 26–65)	51.63 (7.61)	51.91 (7.51)	51.27 (7.74)	1.48
*Psychological wellbeing*				
Tennessee Self – Concept (min/max: 44–100)	80.84 (11.98)	81.79 (11.75)	79.58 (12.17)	3.26***
Beck’s Hopelessness Scale (min/max: 0–16)	4.20 (2.97)	3.96 (2.90)	4.53 (3.04)	−3.41***
Rosenberg Self Esteem Scale (min/max: 16–40)	34.00 (4.57)	34.20 (4.53)	33.75 (4.63)	1.62

*p ≤ .05; **p ≤ .01; ***p ≤ .001

#### Prevalence of depressive symptoms

As presented in Table 3, scores on the BDI scale were divided into four levels of depressive symptoms (minimal, mild, moderate and severe symptoms). In our sample, over one third (31.83%) of participants reported mild symptoms, 29.68% reported moderate symptoms and 16.35% reported severe symptoms. Within age groups, more younger adolescents than older adolescents reported minimal depressive symptoms (22.7% versus 21.4%) and mild symptoms (34.68% versus 28.04%). On the other hand, more older adolescents than younger adolescents reported moderate (33.03% versus 27.16%) and severe symptoms (17.53% versus 15.46%).

**Table 3:** Prevalence of Depressive Symptoms Across Age groups

Depressive Symptoms	Total Sample (*N* = 1260) %(n)	Younger Adolescents: 14–15 years (*n* = 718) %(n)	Older Adolescents: 16–17 years (*n* = 542) % (n)
Minimal	22.14 (279)	22.70 (163)	21.40 (116)
Mild	31.83 (401)	34.68 (249)	28.04 (152)
Moderate	29.68 (374)	27.16 (195)	33.03 (179)
Severe	16.35 (206)	15.46 (111)	17.53 (95)

#### Predictors of depressive symptoms

Results from hierarchical regression analyses are presented in Table 4. Predictors were entered into the model as a block of predictors. In model 1, we control for participants’ sociodemographic and household characteristics (age, orphanhood type, primary caregivers, household size and family assets). In model 2 we add family and social support factors (family cohesion, family and care relationship, perceived child-caregiver support and social support from multiple sources). In model 3 we add measures of psychological wellbeing (self-concept, self-esteem and hopelessness). Age, orphanhood status and family assets were associated with depressive symptoms. Specifically, being an older adolescent girl (16–17 years) was associated with higher levels of depressive symptoms (B = 1.15, 95% CI = 0.02, 2.28, *p* ≤ .05). On the other hand, having both parents alive, i.e. non-orphaned adolescent (B = -1.99, 95% CI = − 3.56, − 0.429, *p* ≤ .01) and higher levels of family assets (B *=* − 0.34*,* 95% CI *=* − 0.51*,* − 0.15, *p* = .001) were both associated with lower levels of depressive symptoms.

**Table 4:** Regression on sociodemographic and household characteristics, family and social support factors, psychological wellbeing

Variable	Model 1: B (95%CI)	Model 2: B (95%CI)	Model 3: B (95%CI)
*Sociodemographic and Household characteristics*			
Age: (*ref:* 14 to 15 *years*)			
16 to 17 years	1.15 (0.02, 2.28) *	0.07(−0.96, 1.09)	− 0.43(−1.41, 0.55)
Orphan hood Status: (*ref: Orphan*)			
Non-orphan	− 1.99(−3.56, − 0.429) **	−1.32(−2.73, 0.09)	−0.89(−2.24, 0.45)
Primary Caregiver: (*ref: Biological parent*)			
Grandparent	0.59(−1.23, 2.42)	0.27(−1.36, 1.91)	0.48(− 1.05, 2.02)
Other relative	−0.21(−2.01, 1.59)	− 0.41(− 2.02, 1.21)	−0.39(− 1.90, 1.13)
Household size			
Number of people in HH	0.29(−0.04, 0.62)	0.14(−0.16, 0.43)	0.09(− 0.19, 0.38)
Number of children in HH	0.01(−0.41, 0.44)	0.08(− 0.29, 0.46)	0.01(− 0.35, 0.38)
Family Assets	− 0.34(− 0.51, − 0.15) ***	−0.14(− 0.30, 0.02)	0.07(− 0.08, 0.23)
*Family and Social Support Factors*			
Family cohesion		−0.0003(− 0.10, 0.09)	0.03(− 0.07, 0.12)
Family care and relationship		−0.38(− 0.51, − 0.24) ***	−0.24(− 0.37, − 0.11) ***
Perceived Child and Caregiver support		−0.12(− 0.19, − 0.03) **	−0.04(− 0.12, 0.04)
*Social support from multiple sources*			
Guardian		−0.31(− 0.46, − 0.17) ***	−0.16(− 0.29, − 0.016) *
Classmate		−0.14(−.30, 0.03)	−0.02(− 0.18, 0.14)
Teacher		−0.13(− 0.28, 0.01)	0.08(− 0.06, 0.22)
Friend/peer		− 0.27(− 0.35, − 0.19) ***	−0.12(− 0.19, − 0.04) **
*Psychological wellbeing*			
Tennessee Self – Concept			−0.28(− 0.33, − 0.22) ***
Beck’s Hopelessness Scale			0.56 (0.35, 0.77) ***
Rosenberg Self Esteem Scale			−0.21(− 0.33, − 0.09) ***
The F-value	4.38***	25.67***	35.37***
R^2^	0.024	0.224	0.356
Adjusted R^2^(df)	0.02 (7)	0.22 (14)	0.35 (17)
Change in R^2^		0.200***	0.132***
N	1260	1260	1106

*p ≤ .05; **p ≤ .01; ***p ≤ .001

When we added family and social support factors in model 2, age, orphanhood status and family assets became non-significant. Family care and relationship (B = − 0.38, 95% CI = − 0.51, − 0.24, *p* = .001), perceived child-caregiver support (B = − 0.12, 95% CI = − 0.19, − 0.03, *p* ≤ .01), social support from a parent/caregiver (B = − 0.31, 95% CI = − 0.46, − 0.17, *p* = .001), and social support from a friend/peer (B = − 0.27, 95% CI = − 0.35, − 0.19, *p* = .001) were all associated with lower levels of depressive symptoms among adolescent girls.

Finally, controlling for measures of psychological wellbeing (model 3), family care and relationship (B = − 0.24, 95% CI = − 0.37, − 0.11, *p* = .001), social support from the parent/caregiver (B = − 0.16, 95% CI = − 0.29, − 0.016, *p* ≤ .05), social support from the peer/friend (B = − 0.12, 95% CI = − 0.19, − 0.04, *p* ≤ .01) remained significant predictors of lower depressive symptoms. In addition, while self-concept (B = − 0.28, 95% CI = − 0.33, − 0.22, *p* = .001) and self-esteem (B = − 0.21, 95% CI: − 0.33, − 0.09, *p* = .001) were both associated with lower levels of depressive symptoms, feelings of hopelessness were associated with higher levels of depressive symptoms (B = 0.56, 95% CI = 0.35, 0.77, *p* = .001).

The model containing sociodemographic and household characteristics accounted for 2.4% (R^2^ = .024) of the variance in depressive symptoms. When we added family and social support factors (model 2), we are able to explain 22.4% (R^2^ = .224) of the variance. The 20-percentage change between model 1 and model 2 was statistically significant (*p* = .001). Adding measures of psychological wellbeing (model 3) allowed us to explain 35.6% of the variance in depressive symptoms (R^2^ = .356). The 13.2 percentage change between model 2 and model 3 was statistically significant (p = .001).

## Discussion

This study utilized baseline data to estimate the prevalence and predictors of depressive symptoms among high school adolescent girls in Uganda. Our findings contribute to the limited literature estimating the prevalence of depressive symptoms–a growing public health concern among adolescents, especially those in low-income countries in SSA. In our sample, the prevalence rates ranged between 16 and 29% (moderate to severe depressive symptoms) and were more pronounced among adolescents 16 years and older. This finding is consistent with previous studies indicating that the severity of mental health disorders, including depressive symptoms continue to rise as children transition through adolescence and into young adulthood [10–12]. The prevalence rates in our sample are consistent with the prevalence rates reported in other studies conducted among high school adolescents in Uganda [73], and indicate high prevalence with significant health, mental health and social implications for adolescents. In particular, adolescents with severe symptoms are at a higher risk of developing later depression [88–90] and other mental health disorders, behavioral health issues, including sexual risk-taking behaviors [31, 91, 92], and poor educational outcomes [93, 94]. Taken together, these findings point to the need for early screening and detection of depressive symptoms to facilitate timely referral to treatment services.

In addition to adolescents’ age, orphanhood status and household assets were associated with depressive symptoms. Although the significance of these two predictors disappear when social support factors are added into the model – signifying possible mediation, these findings are worth discussion. Specifically, having both parents alive was associated with lower levels of depressive symptoms –in an ideal supportive environment, this points to protective role of both parents against depressive symptoms [95]. Indeed, compared to nonorphans, orphaned adolescents are more likely to report poor mental health outcomes, mainly due to psychological distress surrounding parental death, stigma and discrimination associated with orphanhood, financial constraints, and the lack of social support from both within the household and the community [96–100]. On the other hand, possession of family assets (such as land, modes of transportation and communication, gardens, farm animals and small business) was associated with lower levels of depressive symptoms. This finding is consistent with findings from our previous studies that document the positive effects of assets and asset accumulation on adolescents’ depression and overall mental health functioning [60, 63, 65, 101, 102] –pointing to the need for incorporating economic strengthening components in programming for vulnerable adolescents and youth in SSA.

Family and social support factors, including family care and relationships, perceived child-caregiver support, as well as support from caregivers and peers were all associated with lower levels of depressive symptoms among adolescent girls. This finding underscores the role of supportive relationships, especially from family members and peers as protective factors against depressive symptoms [47–50, 67, 103]. However, in our sample, family cohesion was not associated with depressive symptoms. It could be that family closeness alone is not enough to help address adolescents’ depressive symptoms. As such, other supportive strategies to enable adolescents deal with stressful situations as they transition through adolescence, including developing trusting relationships, respectful and open child-caregiver communication, may be important. Indeed, one of the intervention components of the study focuses on strengthening family relationships to address mental health challenges and sexual risk-taking behaviors among adolescent girls [75]. We hope that analysis of post intervention data will reveal significant improvements, not only in family functioning, but also in the overall mental health functioning of study participants.

Finally, adolescents’ psychological wellbeing, including self-esteem, self-concept, as well as lower levels of hopelessness, were all associated with low levels of depressive symptoms. Indeed, the model controlling for these measures explained the greatest variance in depressive symptoms. Consistent with other studies conducted elsewhere [38–42], it is critical to develop and/or strengthen these mechanisms among adolescents earlier on to build resilience and lessen the impact of risk factors associated with depressive symptoms and facilitate a smooth transition through adolescence.

### Limitations

These findings should be carefully interpreted in light of the following limitations. First, the Beck Depression Inventory serves only as a screening tool for depressive symptoms. Scores are not meant to be interpreted as a diagnosis –as this requires further assessment. Second, we utilized data from one group– adolescent girls without a comparable group of adolescents. However, we do know from prior literature that the prevalence of depressive symptoms tend to be higher among adolescent girls and women compared to adolescent boys and men. Third, adolescents’ age range was not broad enough to deduce robust gender differences. Fourth, all data was self-reported by adolescents –which may suffer from social desirability. Finally, we report baseline data only and are unable to make any causal inferences. Given that the study is still in the intervention implementation phase, post intervention data will be analyzed in the near future.

## Conclusions

Study findings contribute to the limited literature estimating the prevalence and correlates of depressive symptoms among adolescent girls in low resource settings in Uganda. We find high prevalence rates of depressive symptoms, ranging between 16 and 29%, and more pronounced among adolescents 16 years and older. These high rates point to the higher risk of developing later depression, if not addressed. Given that depressive symptoms tend to increase during later adolescence, our findings point to the need for early screening and detection to facilitate timely referral to support and treatment services. Family support factors and adolescents’ psychological wellbeing were associated with low levels of depressive symptoms. These findings may inform the development and incorporation of gender-specific mental health components -especially those that target family support, economic strengthening, as well as psychological wellbeing among adolescents, living in low-resource communities in SSA.

## Data Availability

The datasets analyzed during the current study are available from the corresponding author on reasonable request.

## Abbreviations

SSA: Sub-Saharan Africa
UCE: Uganda Certificate of Education
CITI: Collaborative Institutional Training Initiative
BDI: Beck’s Depression Inventory
RSES: Rosenberg Self-Esteem Scale
BHS: Beck Hopelessness Scale
TSCS: Tennessee Self-Concept Scale
